# Adolescent idiopathic scoliosis (AIS), environment, exposome and epigenetics: a molecular perspective of postnatal normal spinal growth and the etiopathogenesis of AIS with consideration of a network approach and possible implications for medical therapy

**DOI:** 10.1186/1748-7161-6-26

**Published:** 2011-12-02

**Authors:** R Geoffrey Burwell, Peter H Dangerfield, Alan Moulton, Theodoros B Grivas

**Affiliations:** 1 Centre for Spinal Studies and Surgery, Nottingham University Hospitals Trust, Queen's Medical Centre Campus, Derby Road, Nottingham, NG7 2UH, UK; 2 University of Liverpool, Ashton Street, L69 3GE, UK; 3 Staffordshire University, Leek Road, Stoke-on-Trent, ST4 2DF. UK; 4 Royal Liverpool Children's Hospital, Eaton Road, Liverpool, L12 2AP, UK; 5 Department of Orthopaedic Surgery, King's Mill Hospital, Sutton Road, Mansfield NG17 4JL, UK; 6 Department of Trauma and Orthopedics, "Tzanio" General Hospital, Tzani and Afendouli 1 st, Piraeus 18536, Greece.co.uk

## Abstract

Genetic factors are believed to play an important role in the etiology of adolescent idiopathic scoliosis (AIS). Discordant findings for monozygotic (MZ) twins with AIS show that environmental factors including different intrauterine environments are important in etiology, but what these environmental factors may be is unknown. Recent evidence for common chronic non-communicable diseases suggests *epigenetic differences *may underlie MZ twin discordance, and be the link between environmental factors and phenotypic differences. DNA methylation is one important epigenetic mechanism operating at the interface between genome and environment to regulate phenotypic plasticity with a complex regulation across the genome during the first decade of life. The word *exposome *refers to the totality of environmental exposures from conception onwards, comprising factors in *external *and *internal environment*s. The word *exposome *is used here also in relation to physiologic and etiopathogenetic factors that affect normal spinal growth and may induce the deformity of AIS. In normal postnatal spinal growth we propose a new term and concept, *physiologic growth-plate exposome *for the normal processes particularly of the *internal environments *that may have epigenetic effects on growth plates of vertebrae. In AIS, we propose a new term and concept *pathophysiologic scoliogenic exposome *for the abnormal processes in molecular pathways particularly of the *internal environment *currently expressed as etiopathogenetic hypotheses; these are suggested to have deforming effects on the growth plates of vertebrae at cell, tissue, structure and/or organ levels that are considered to be epigenetic. New research is required for chromatin modifications including DNA methylation in AIS subjects and vertebral growth plates excised at surgery. In addition, consideration is needed for a possible *network approach *to etiopathogenesis by constructing AIS *diseasomes*. These approaches may lead through screening, genetic, epigenetic, biochemical, metabolic phenotypes and pharmacogenomic research to identify susceptible individuals at risk and modulate abnormal molecular pathways of AIS. The potential of epigenetic-based medical therapy for AIS cannot be assessed at present, and must await new research derived from the evaluation of epigenetic concepts of spinal growth in health and deformity. The tenets outlined here for AIS are applicable to other musculoskeletal growth disorders including infantile and juvenile idiopathic scoliosis.

## Introduction

The principal aim of this paper is to examine the etiopathogenesis of adolescent idiopathic scoliosis (AIS) from the standpoint of epigenetics. To our knowledge this has not previously been addressed. Epigenetics, a relatively recent field now vast and vigorous, evaluates factors concerned with gene expression in relation to environment, disease, normal development and aging, with a complex regulation across the genome during the first decade of life. Butcher and Beck [1] describe epigenetics as follows:

"Although environmental measures are logical covariants for genotype-phenotype investigations, another non-genetic intermediary exists: epigenetics. Epigenetics is the analysis of somatically-acquired and, in some cases, transgenerationally inherited epigenetic modifications that regulate gene expression, and offers to bridge the gap between genetics and environment to understand phenotype. The most widely studied epigenetic mark is DNA methylation. Aberrant methylation at gene promoters is strongly implicated in disease etiology, most notably cancer."

There is controversy relating to the definition of epigenetics which we outline. Taking the broad definition, a view of AIS etiopathogenesis and normal spinal development is presented from an epigenetic standpoint, predicated on a model for other diseases.

Research into the causation of adolescent idiopathic scoliosis (AIS) draws heavily from mechanical and biological disciplines, but still lacks an agreed theory of etiopathogenesis [2,3]. Genetic factors are believed to play an important role in the etiology of AIS with considerable heterogeneity [2,4,5]. Hence treatment is empirical and not based on sufficient understanding of etiology to support the current mechanically-based therapy [6]. The research problem is complicated by the suspicion that AIS may result not from one cause, but several that interact. Genetic, and now genomic, research on AIS has not yet provided the therapeutically-required etiologic understanding. In other diseases and particularly diseases of developmental origin [7-13] and late-onset chronic non-communcable diseases (NCDs) [14-22], research on the role of environmental factors and *epigenetics *after a slow start [23] has exploded in the last decade [1,17,18,24-32]. Not so for AIS research where, except for monozygotic twin studies and very recent mentions on the net [33,34], there are only sporadic reports suggesting that environmental factors are at work in etiology.

Genotype-environment (GxE, nature/nurture) interactions are being extensively researched in human growth [35-40], behavioural studies [41-43], early-life conditions [7-13,44,45], placentation [29] and gastrointestinal diseases [46-48]. The increasing incidence of idiopathic club foot in Denmark and Sweden has led to the speculation that factors associated with population density namely, environmental stress (traffic pollution, noise) and stress of urban living (misuse of tobacco, alcohol, drugs), could be reasons for this epidemiological change [49].

DNA methylation (DNAm) is an important epigenetic mechanism operating at the interface between genome and environment to regulate phenotypic plasticity with a complex regulation across the genome during the first decade of life [50]. Recent data suggests that epigenetic responses including DNAm is involved not only in cellular differentiation but also in modulation of genome function in response to signals from the various environments [45]. The window of developmental plasticity extends from preconception to early childhood and is exerted particularly during life-history phase transitions [13]. Developmental origins of health and disease and life-history transitions are purported to use placental, nutritional, and endocrine cues for setting long-term biological, mental and behavioural strategies in response to local physical, biological and/or social conditions [13,45,51].

*Epigenetics *is now generally defined as information heritable during cell division but not contained within the DNA sequence itself [14]. There are three major ways organisms alter their DNAs inherited messages: enzymes methylate DNA to modulate transcription; histone modifications and nucleosome positioning to induce or repress target sequences; and non-coding small RNAs (including microRNAs and short interfering RNAs) which attach themselves to messenger RNA to modify the expression of specific genes [10,46,52,53]. DNA-cytosine methylation is a central epigenetic modification that has essential roles in cellular processes including genome regulation, development and disease [54]. According to Cropley et al [55] epigenetic mechanisms provide multicellular organisms with a system of normal gene regulation that silences portions of the genome and keeps them silent as tissues differentiate. Long-term silencing can be reprogrammed by demethylation of DNA which starts afresh in each generation in germ cells and early embryos through which effects on nutrition *in utero *may influence health in later life [56-58] (Appendix I).

Errors in this complex system termed *epimutations *arising from environmental and stochastic (random) events, can give rise to abnormal gene silencing, that may result in a great deal of phenotypic variation and common disease, At present, there are only a handful of clear examples; but importantly this can occur in the absence of any underlying genetic defect [59]. Alterations in the epigenetic status can be directly modified by various environmental insults or maternal dietary factors [44,60]. *Epigenetics *helps to explain the relationship between an individual's genetic background, environment, aging, and disease [17]. Sex differences in epigenetic processes may alter the risk or resilience to develop a particular disorder [61]. Increased understanding of epigenetic-disease mechanisms could lead to innovative diagnostic tests and disease-risk stratification to targeted intervention and therapies [16,46].

The *Human Epigenome Project (HE) *and other epigenomic projects [62-66] are evaluating e*pigenetics *in developmental origins of human disease [9,11], and for musculoskeletal disorders in bone development [67-69] and dysmorphology [70].

**Figure 1:**
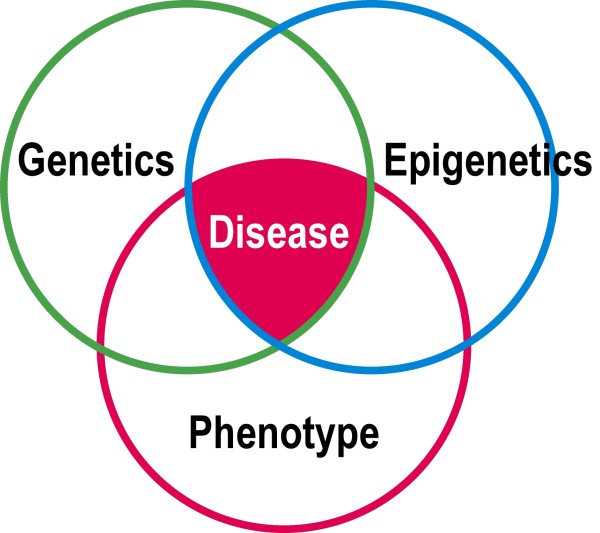
**Venn diagram showing relationship between genetic variation, epigenetic variation and disease simultaneously (Modified from Feinberg **[18]).

Apart from the emerging role of epigenetic mechanisms in the etiology of neural tube defects [60], Prader-Willi syndrome [71,72], and the recent theoretical interpretations of Burwell and colleagues [73-76] and McMaster [77], *epigenetics *does not figure in any causal analysis of postnatal normal spinal growth, or in the etiopathogenesis of AIS (Figure 1), This reflects current scientific opinion that genetic rather than environmental factors determine the etiology of AIS in accordance with the *genetic variant hypothesis of disease *[17,78] (Appendix II).

In this paper we briefly evaluate postnatal normal spinal growth and the etiopathogenesis of AIS in relation to the epigenetic explosion and the terminologic disagreements; the latter arise from the different requirements of geneticists, molecular biologists, developmental biologists and pathogetieticists. Our interpretation for AIS attempts to overcome these difficulties. It is predicated on the premise that in all scolioses, idiopathic, and secondary, spinal deformity cannot occur without normal vertebral growth-plate function being compromised ultimately in three dimensions by abnormal processes. This focus on vertebral growth does not imply that asynchronous ribcage growth [79-83], intervertebral discs and vertebral bone may not contain factors in AIS pathogenesis. The tenets outlined here for AIS are applicable to other developmental growth disorders including infantile and juvenile idiopathic scoliosis.

The aims of this paper are:

1. To review sporadic reports suggesting that environmental factors are involved in AIS etiology.

2. To note that the risks of developing late-onset chronic non-communicable diseases (NCDs) including cancer, diabetes, cardiovascular disease, respiratory disease, obesity and schizophrenia, are attributed to genetic and environmental factors.

3. To discuss the meaning of the word *exposome*. Currently, it refers to the totality of environmental exposures, exogenous and endogenous from conception onwards, some of which lead to occupational health problems.

4. To use the word *exposome *also in relation to physiologic and etiopathogenetic factors that respectively affect normal spinal growth and may induce/promote the deformity of AIS.

5. To suggest that the harmful effects produced by exposome factors leading to dysfunction involve interference with normal cellular processes and molecular pathways in cells, tissues, structures and organs.

6. To define *epigenetics*, its origin and two current meanings, *modification *and *interactions*.

7. To outline *epigenetics *in relation to normal embryonic development, involving *epigenetic modification and interactions*.

8. To present a causal analysis of the normal postnatal normal spinal growth putatively involving *epigenetic interactions *and *modification*.

9. To apply a new collective term and concept, *physiologic growth-plate exposome*, to the mainly endogenous (internal) environmental processes that affect normal spinal growth.

10. To present a causal analysis of abnormal deforming postnatal spinal growth in AIS, putatively involving *epigenetic interactions *and *modification*.

11. To apply a new collective term and concept, *pathophysiologic scoliogenic exposome *to abnormal processes in developmental pathways particularly of the *internal environment *that have putative epigenetic deforming effects on the growth plates of vertebrae at cell, tissue, structure and organ levels, and currently expressed as etiopathogenetic hypotheses.

12. To consider that the pathogenesis of AIS may involve *a one--hit to multi-hit model*.

13. To suggest research on chromatin modification including DNA methylation (DNAm) which plays an important role in gene expression, using tissues from AIS subjects including vertebral growth plates excised at surgery

14. To touch on *network medicine *and consider a network approach to AIS etiopathogenesis by constructing AIS *diseasomes*.

### Environmental risk factors for adolescent idiopathic scoliosis (AIS)

Thirty years ago Wynne-Davies [84] examining the etiology of some common skeletal deformities including infantile idiopathic scoliosis, concluded that all are likely to have a common multifactorial genetic background associated with differing intrauterine or postnatal environmental factors. Most authors state that genetics stipulates the course of adolescent idiopathic scoliosis (AIS). In the last 20 years, sporadic reports have suggested environmental factors are involved in the etiopathogenesis and phenotypic expression of AIS. The evidence is outlined here together with an interpretation of AIS pathogenesis and the environment by some workers [85-87].

#### Monozygotic (MZ) twins and spinal radiology in AIS

MZ twins have a significantly higher concordance for AIS than dizygotic twins, with scoliosis curves in MZ twins developing and progressing together. Based on these data, Kesling and Reinker [88] concluded there is strong evidence for a genetic etiology for AIS, and familial idiopathic scoliosis [89,90].

MZ twins have been used to demonstrate the role of environmental factors in determining complex diseases and phenotypes, but the true nature of the phenotypic discordance remains poorly understood [24,50]. In AIS, concordance rates in MZ twins are 0.73-0.92 [88,91,92] with lower figures of 0.13 and 0.10 reported respectively from the Danish Twin Registry [93] and Swedish Twin Registry [94]. These findings are quite surprisingly different, and suggest that variation in diagnostic criteria is important in the results of these studies [Armour J personal communication]. The Swedish Twin Registry study revealed a unique environment effect of 0.60 [94] suggesting environmental factors are important in the etiology of AIS from different intrauterine environments [88]. In 32 MZ twins, van Rhijn et al [95] found several parameters - gender, direction of convexity, apical level and kyphotic angle - were determined more by genetic factors than the lateral Cobb angle, suggesting that curve severity may be affected by the environment. Mirroring of curves was found in each of two MZ twin sets with idiopathic scoliosis [88,96]. In another MZ twin pair concordant for AIS, the twins had different apical levels, curve magnitudes, and age at detection which stress the importance of environmental (non-genetic) factors in etiopathogenesis [97].

Concordance rate of less than 100% in MZ twins for AIS may be explained not only from environmental influences but also by other factors including, uneven cytoplasmic cleavage of the fertilized egg - thought to cause the scoliosis mirroring of one twin pair, mutation after fertilization causing a genotype mosaic [88], differences in placentation, amniotic sac, and vascularization of separate cell masses [24]. In MZ twins with *congenital scoliosis*, environmental factors are reported to play a leading role in the development of the condition [98].

According to Fraga et al [24]* epigenetic differences *may underlie MZ twin discordance for common diseases, and represent the link between environmental factors and phenotypic differences. The patterns of epigenetic modification of twin pairs diverge as they become older and their lifestyles become distinct reflecting accumulated exposure to a wide range of external and internal factors including environmental factors such as physical (and perhaps mental) activity, diet, drink, smoking and other habits [99]. They referred to the phenomenon as "*epigenetic drift*" and associated it with the aging process [100].

#### A food and growth connection?

A sudden increase in the incidence of idiopathic scoliosis in Jamaica after 1965 was evaluated by Golding [101]. Attention was directed to endocrine additives used to promote the growth of livestock and the meat-to-food conversion in cattle and broilers. Taking Into consideration the 10-year delay in the onset of the idiopathic scoliosis, this fitted remarkably well with increase frequency of the scoliosis which occurred up to 1983 and its decline since.

#### Nutrition in the etiology of idiopathic scoliosis (IS)

A review of American and European articles from 1955-1990 evaluated nutrition as an environmental factor in the etiology of idiopathic scoliosis [102]. These authors concluded:

"...there is at least an anecdotal association of IS with poor nutrition, there is strong evidence from an animal model and there is a partial understanding of the biochemical mechanisms explaining nutrition as an etiological factor. Given the fact that nutrition is an environmental factor which can easily be changed, further investigation of the link between nutrition and IS in humans is warranted."

#### Relative osteopenia and life-style factors

Studies on Chinese girls with AIS have revealed relative osteopenia [113,114] suggesting a contribution from life-style factors including nutrition, diet, calcium, vitamin D intake and exercise level [[Bibr B2]]

Good dietary practices and optimal nutritional status are known to promote growth and tissue development, as well as disease prevention [39,53,103,104]. Nutritional epigenetics has emerged as a novel mechanism underlying gene-diet interactions [104] with the strongest evidence for transgenerational inheritance coming from the survivors of the Dutch Hunger Winter [105]. Dietary modification can have a profound effect on DNAm and genomic imprinting [16,106], with plant-derived microRNAs entering the bloodstream [53]. A major focus of research on dietary influences on epigenetic status has been on nutrition *in utero*, because the epigenome is probably malleable particularly during this life-course window [60,107,108], and because epigenetic marking by early exposures is a compelling mechanism underlying effects on lifelong health [108,109]. DNAm depends on dietary methionine and folate, both of which are affected by the nutritional state [14,17,110]. Ford et al [108] have published a Table summarizing specific dietary components with effects on DNAm; these include methyl donors, bioactive polyphenols, zinc, selenium,and vitamin A. In AIS, prevention by diet is discussed speculatively [111,112], and on the net [34].

#### Physical activities of patients with AIS

McMaster et al [115,116] reported AIS to be negatively associated with participation in dance, skating, gymnastics/karate and horse riding classes. They asked the question: Do children who develop AIS have a long-standing proprioception defect which makes them less likely to participate in sporting activities? If so, by encouraging sport and increasing proprioceptive abilities common to all joints [117] might make those at risk less likely to develop spinal asymmetry.

#### Geographic latitude and the prevalence of AIS

In a review of peer-reviewed published papers, Grivas et al [118] found that a later age at menarche is associated with a higher prevalence of AIS. The prevalence decreases as geographic latitude approaches the equator, suggesting a possible role for environmental factors in the pathogenesis of AIS in girls. A slight delay of menarcheal age in northern countries by lengthening the period of spine vulnerability to etiologic factors, was suggested as a pathogenetic mechanism.

#### Maternal age and socio-economic status

In a study of 404 children with idiopathic scoliosis predominantly from New York State there was an excess of *propositi *born to mothers at ages 30-39 years [119]. Wynne-Davies [89,120] reviewing 94 children with AIS found in girls and boys, maternal but not paternal age was significantly in excess of normal. In a Swedish study of perinatal and environmental aspects of 551 adolescent patients with thoracic idiopathic scoliosis, maternal age was higher, birth weight normal, scoliosis commoner in higher socioeconomic groups, and the illegitimacy rate half that expected [121]. These findings from the USA, Scotland and Sweden are consistent and reveal increased maternal age as a risk factor for AIS, suggesting maternal factors can predispose to it. The intra-uterine environment is crucial in programming the fetus for various health and disease outcomes throughout life [44].

#### Heated indoor swimming pools infants and delayed epigenetic effects

In a case-control study in Scotland, McMaster et al [115,116,122] reported a statistically significant correlation between the introduction of infants to heated indoor swimming pools and the development of AIS. A neurogenic hypothesis was formulated to explain how toxins produced by chlorine in such pools may act on the infant's immature central nervous system; through vulnerability of the developing brain to circulating toxins and *delayed epigenetic effects *with the bony trunk deformity of AIS not becoming evident until adolescence [77]. There may be many such environmental factors acting in the first year of life to initiate AIS and differing around the world, with one environmental factor involving heated indoor swimming pools being detected in Scotland [77]. Whatever effects the neurotoxic products may have on the immature brain, the process of puberty with its increased growth velocity is suggested to play a role in the delayed phenotypic expression of AIS [77].

#### Non-surgical treatments for AIS

Publications on environmental effects induced in the spine by physical exercises and brace treatments will not be considered here.

#### Hypothesis of developmental instability for scoliosis

Speculation that genetic and environmental factors are involved the etiopathogenesis of idiopathic scoliosis [123,124] was developed by Goldberg and colleagues [85-87] who suggested that scoliosis is caused by environmental stress causing *developmental instability*:

*"....scoliosis is not a disease or group of diseases but a symptom or sign of environmental stress, significant enough to overwhelm the intrinsic stability of the morphological genome. As such, there is no specific etiology but a large number of precipitating stressors...."*[87].

Such environmental factors could be hormonal, nutritional, alcohol, smoking, viruses, drugs, medicaments, radiation, maternal reactivity to male-specific features of the fetus, hypoxia during birth [111], factors associated with population density [49], toxins in heated indoor swimming pool [77], and lack of physical activity. [115,116].

The *hypothesis of devevlopmental instability *applied to scoliosis is contained within both the *developmental origins of health and disease concept *(*DOHaD*) [9,12,13] and the *common disease genetic and epigenetic model *for late-onset chronic non-communicable diseases *(CDGE) *[14,17]. Both the *DOHaD and CDGE models *for disease invoke epigenetic mechanisms [125] (Appendix III).

### Chronic diseases, external and internal environments, phnotypic plasticity, exposome

The risks of developing late-onset chronic non-communicable diseases (NCDs) including cancer, diabetes, cardiovascular disease, respiratory disease, obesity and schizophrenia, are attributed to both genetic and environmental factors; 70-90% of disease risks are thought to be due to differences in environments [19,126]. Hanson et al [20] comment that progress in this field has been slow due to an excessive emphasis on fixed genomic variations (hard inheritance) as the major determinants of disease susceptibility. However, new evidence demonstrates the much greater importance of early-life developmental factors, involving epigenetic processes and 'soft' inheritance in modulating an individual's vulnerability to NCDs. According to Rappaport and Smith [19] epidemiologists increasingly use genome-wide association studies (GWAS) to investigate NCDs, and rely on questionnaires to characterize "environmental exposures". The risk factors for NCDs include smoking, unhealthy diet, lack of physical activity and alcohol abuse [21,22].

#### Exposome

The word *exposome *[127] refers to the totality of environmental exposures from conception onwards that create dysfunction which in some individuals leads to occupational health problems [128]. The *exposome *comprises exogenous and endogenous factors: exogenous factors in the *external environment *- chemicals (toxicants) entering the body from air, water and food, *eg *diet, food supplements, life style, drugs, chemicals; and endogenous factors in the *internal environment *- chemicals produced in the body *eg*, oxidative stress, lipid peroxidation [111,112,129], gut flora, and other natural processes, including biomarkers. Any harmful effects leading to dysfunction evidently interferes with normal cellular processes and molecular pathways in cells, tissues, structures and organs with the individual response to environmental factors being genetically influenced [129]. There are likely to be critical periods of exposure in development with vulnerability to the exposome [126]. Human evolution through natural selection has involved reduced exposure to challenges from external and internal environments [130].

### Definitiions: epigenetics, origin and its recent double meaning in health and disease

*Epigenesis *in biology describes the morphogenesis and development of an organism with organ systems that are not preformed.

#### Epigenetics

The word *epigenetics *was coined by Waddington [131] to link the two fields of developmental biology and genetics, hitherto considered as separate disciplines [132-134]. There are now several definitions of *epigenetics *[14,18,25,135-140] (Appendix IV). Waddington's broad view of *epigenetics *fell out of favor in modern biology to be replaced with a much narrower one defining *epigenetics *as:

*" ....modifications of the DNA or associated proteins, other than DNA sequence variation, that carry information content during cell division." *[10,14,17,25,29].

These changes result from chemical alterations to DNA or associated histone proteins termed *epigenetic modification*, occurring in health and disease from stochastic (random) and environmental factors modulating transcription from chromatin [29,141]. The best known example of epigenetic modification is DNA methylation (DNAm). Cell type-specific DNAm patterns are established during mammalian development and maintained by tissue-specific gene expression in adult somatic cells [142]. DNAm plays an important role in programming gene expression, including the regulation of changes in gene expression in response to aging and environmental signals [104,143]. Loss of methylation, which may result from enzymic mechanisms will lead to heritable abnormalities in gene expression, and these may be important in oncogenesis and aging [104,142-144].

*Methylome *refers to the genome-wide state of DNAm [18]. Advances in sequencing methods have allowed measurement of the first complete genome-wide *DNAm map *(*methylome ) *in human cells [54,145,146].

*Epigenotype *refers to information in a cell that is maintained through mitosis and/or meiosis but does not involve DNA sequence itself [14].

*Epigenome*, is the sum total of all the epigenetic information in a cell; there are as many *epigenomes *as there are cell types [26]. It has been likened to an archive of the prenatal environment [44]. The *epigenome *parallels the word *genome*, and refers to the overall epigenetic state of a cell. Although all (nucleated) human cells effectively contain the same genome (and therefore the same genetic instruction sets), they contain very different *epigenomes *depending upon cell type, developmental stage, sex, age, environmental cues, various other parameters and maintain different terminal phenotypes [147,148]. *Epigenomics *refers to the genome-scale analysis of epigenetic marks [18].

*Epimutations and disease*. According to Martin et al [59], epigenetic silencing is a pervasive mode of gene regulation in multicellular animals. Epigenetic silencing is not irreversible and requires active maintenance. This requirement for active maintenance of epigenetic states, creates the potential for errors on a large scale. When epigenetic errors - or *epimutations *- activate or inactivate a critical gene, they may cause disease. They define *epigenetic disease *as: *"...one caused by stable alteration in the epigenetic state of a gene (epimutation) without any contributory genetic mutation."*

*Epigenetic modification and interactions*. Very recently, some workers have returned to using Waddington's more inclusive definition to bridge more fully the gap between genotype and phenotype, introducing the term *epigenetic interactions *[138-140,149]. This concept does not entirely accord with the view that an epigenetic system should be heritable, self-perpetuating and reversible [141]. Whether or not the term *epigenetics *retains its original meaning or becomes restricted to chromatin modification remains to be seen [149]. Taken together both terms, *modification *and *interactions*, enrich and broaden our view of development, evolution and disease [149].

How may these new *epigenetic *concepts of *modification *and *interactions *be related to normal development?

### Epigenetics - relevance for normal development

**Figure 2:**
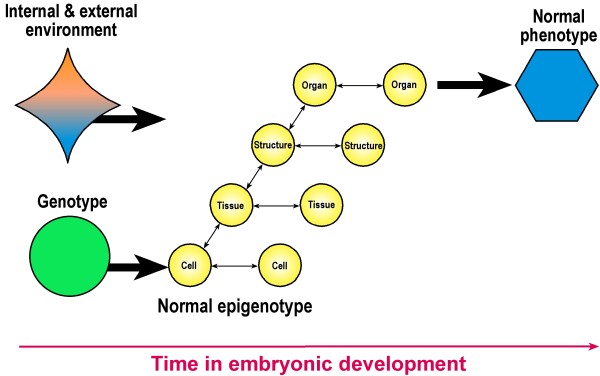
**Normal embryonic development over time initiated by genetic factors (green) and environmental factors (internal blue, external orange ) leading to the normal phenotype (blue)**. Small arrows represent epigenetic interactions occurring at call, tissue, structure and organ levels (Modified from Jamniczky et al [140]).

*Developmental biology and embryonic development*. Figure 2 shows that in normal embryonic development, epigenetic changes may occur at each of cell, tissue, structure and organ levels. A cell's environment, location and surroundings provide epigenetic factors that influence the cell's identity and activities [119] as it rolls down Waddington's metaphorical epigenetic landscape [150]. Francis [151] states that the fate of each cell is largely determined by its position in the embryo and the nature of its neighbour cells with which it chemically interacts; this is termed *patterning *in skull development [74,75,138,139]. These intercellular interactions influence the environment within the cell, which in turn influences which genes are epigenetically activated or inactivated. At organ level, the growing brain influences the development of the skull presumably involving mechanical interaction (mechanotransduction) as an epigenetic interaction [137,140].

#### Epigenetic modifications and regulation

**Figure 3:**
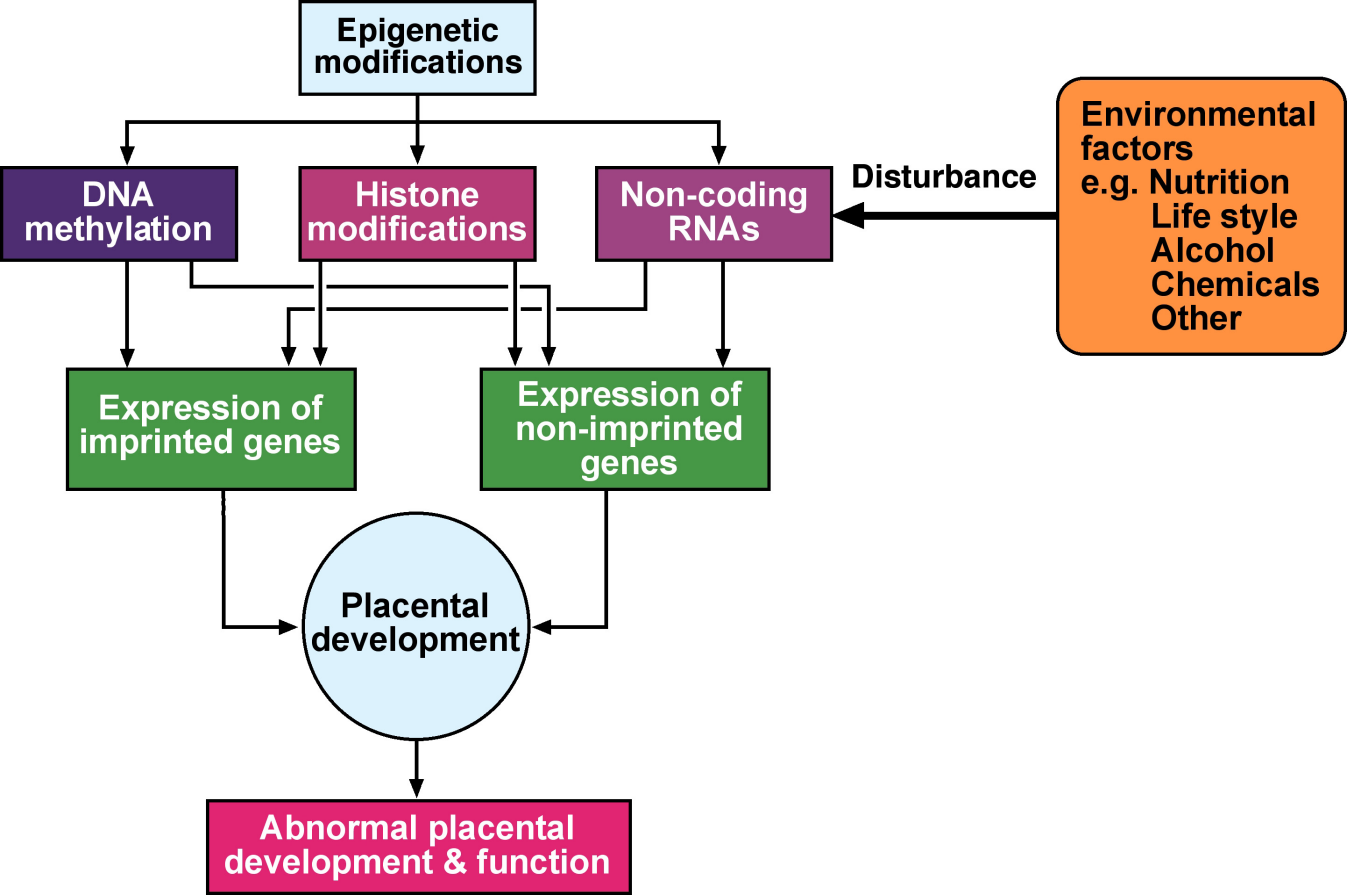
**Epigenetic modifications in placental development and possible consequences of its disturbance which can be caused by environmental factors**. X-chromosome inactivation (lyonization) is not shown [152-154] (Modified from Nelissen et al [29]).

Figure 3[152-154] shows predominant epigenetic modifications: DNA methylation (DNAm) modifications to histones, non-coding RNAs and parent-of-origin imprinting for placental development. (Appendix V)[155-172] [see Figure 2, reference [10] and [29,46,141,153]].

#### Epigenetic interactions

**Figure 4:**
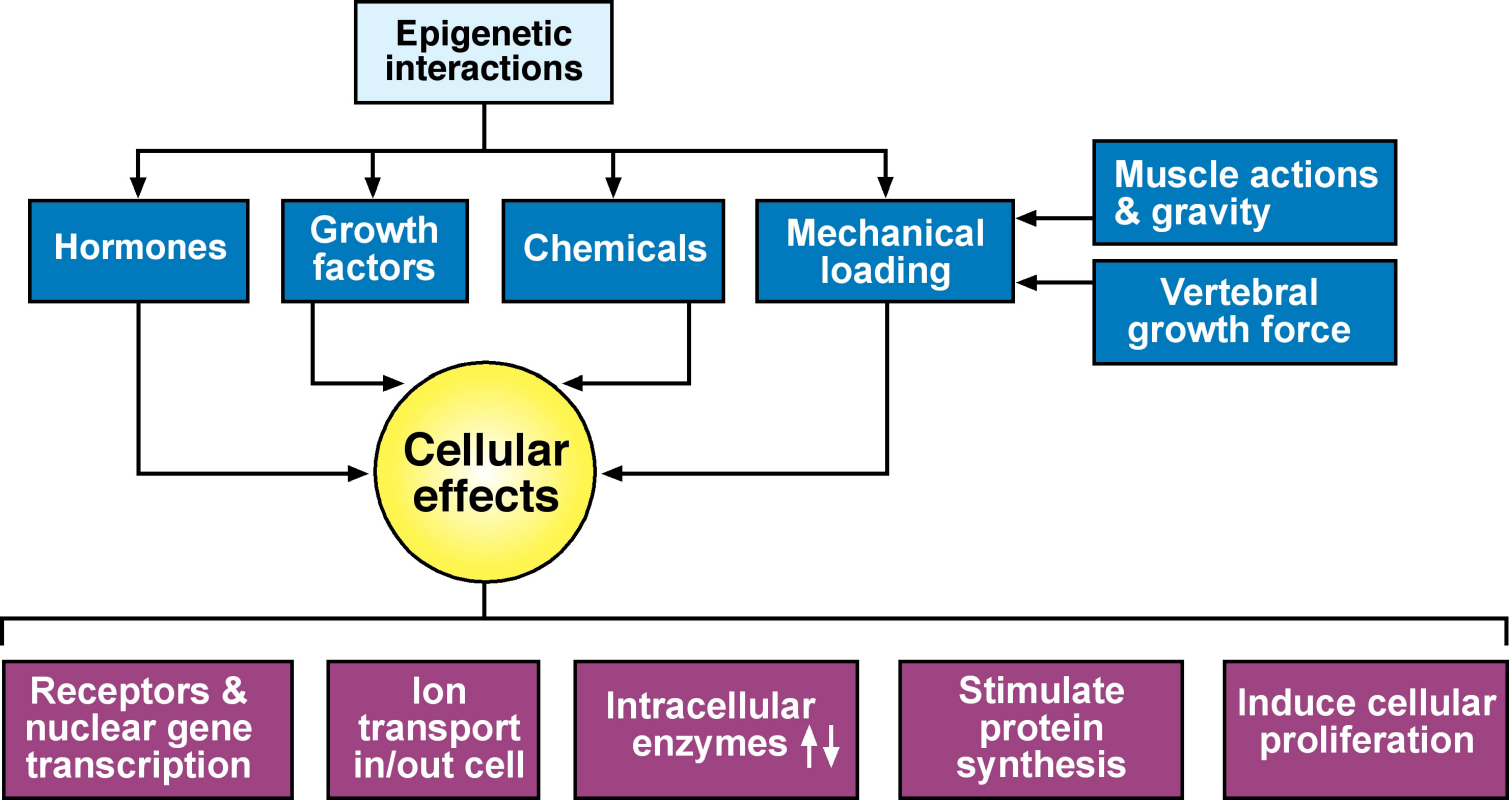
**Epigenetic interactions as applied putatively to normal vertebral growth**. (Drawn from descriptions mostly of Herring [136] and Lieberman [138]).

Figure 4 shows epigenetic interactions for normal vertebral growth constructed mostly from the descriptions of Herring [136,137] and Lieberman [138,139], and for chemicals from other workers [173-175].(Appendices IV & VI).

How may *epigenetic modification and interactions *relate to normal postnatal spine growth?

### Normal postnatal spine development - physiologic growth-plate exposome

**Figure 5:**
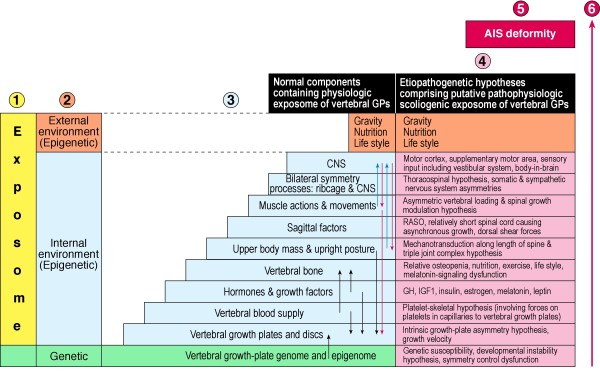
**Putative genetic and epigenetic approach to causal factors affecting vertebral growth plates (GPs) in health and AIS pathogenesis (CNS = central nervous system)**. Moving from left-to-right in columns: 1) Exposome (yellow ); 2) external environment (epigenetic, orange), internal environment (epigenetic, blue) and genetic (green); 3) factors controlling normal vertebral growth, genetic (green), internal environment (blue) and external environment (orange) containing the *physiologic growth-plate exposome; *these factors are considered to cause epigenetic changes (follow vertical arrows) in normal structures and contribute to the epigenome of vertebral growth plate cells; 4) etiopathogenetic hypotheses for AIS containing the *pathophysiologic scoliogenic exposome *(pink) and genetic susceptibility (pink); 5) the resulting AIS deformity (red); 6) the long vertical red arrow to the right represents craniocaudal pathophysiologic components affecting the trunk over time leading to the AIS deformity. (Adapted to normal spinal growth and AIS pathogenesis from the multihit pathogenetic model for inflammatory bowel disease of Maloy and Powrie [47] and the genetic/epigenetic model for common human diseases of Bjornsson et al [14]).

Figure 5 shows factors that affect growth of the normal spine through vertebral growth plates. They are shown as three groups: genetic, internal environment, and external environment. Besides genetic control, the growth of normal vertebral growth plates is influenced by factors mainly within the *internal environment; *these include hormones [133,136,176,177], growth factors [176] chemicals [173-175] and mechanical forces [133,136,137,178,179]; the latter are created by the vertebral growth force [136,180], gravity (a weak force [181-183], upright posture and muscular contractions under central nervous system control acting against gravity. *External environmental factors *include gravity, nutrition and lifestyle.

We propose the term, *physiologic growth-plate exposome *for the mainly internal normal environmental processes that affect normal spinal growth. The effects of this *physiologic exposome *on vertebral growth plates is viewed as epigenetic. This will involve *epigenetic interactions*. How much *epigenetic modification *is involved is unknown.

### Adolescent idiopathic scoliosis - pathophysiologic scoliogenic exposome

Figure 5 shows etiopathogenetic hypotheses for AIS which express abnormality(ies) in normal developmental pathways of one or more of the normal internal environmental processes. Whether one (*one-hit model) *or several (*multi-hit model*) abnormalities are involved in pathogeenesis for different individuals with AIS is unknown. We propose a new term and concept, *pathophysiologic scoliogenic exposome *be applied to abnormal processes in normal developmental pathways particularly of the *internal environment *that have putative epigenetic deforming effects on the growth plates of vertebrae [184-191] at cell, tissue, structure and organ levels, and currently expressed as etiopathogenetic hypotheses (Appendix VII) [192-242]. This will involve epigenetic interactions. How much *epigenetic modification *is involved is unknown.

**Figure 6:**
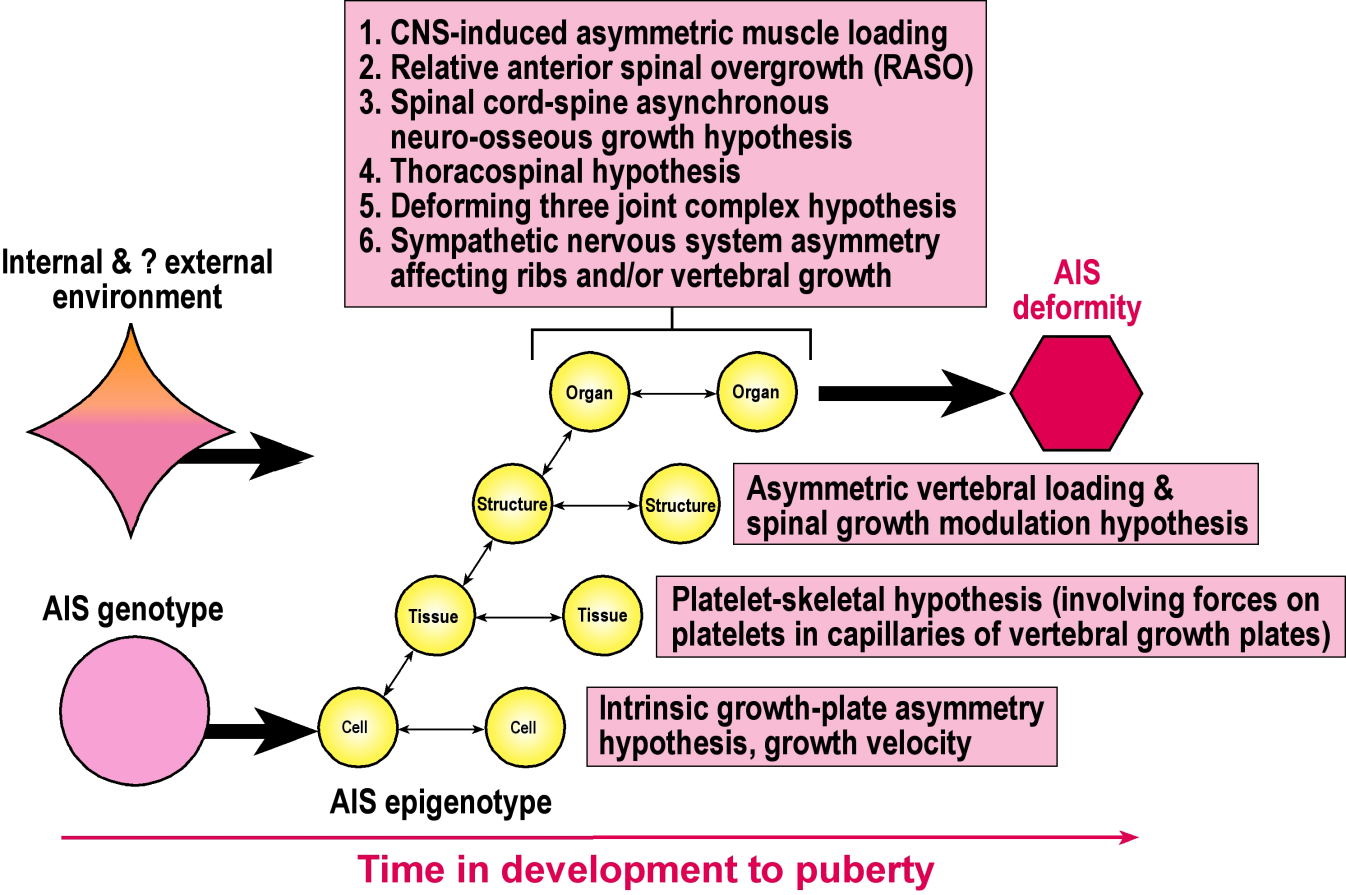
**Postnatal development of AIS in the spine over time to puberty initiated by genetic factors (pink) and environmental factors (internal pink for AIS, ? external orange) leading to AIS deformity (red)**. Etiopathogenetic hypotheses acting at cell, tissue, structure and organ levels are linked to putative epigenetic mechanisms affecting vertebral growth plates. For example, in the asynchronous spinal neuro-osseous growth concept [208-211] subclinical tether of a relatively short spinal cord causes a lordoscoliotic maladaption of the spine leading to relative anterior spinal overgrowth (RASO) and the AIS deformity. These adaptive changes in the anterior spinal column are viewed as occurring at cell, tissue and organ levels, and resulting from mechanically-induced effects in vertebral growth plates from *epigenetic interactions *and/or *epigenetic modification in *vertebral growth-plates (Adapted from Jamniczky [140]).

Figure 6 shows etiopathogenetic hypotheses acting at cell, tissue, structure and organ levels linked to possible epigenetic mechanisms affecting vertebral growth plates, possibly in a diverse network of developmental pathways [243].

#### Trunk velocity of growth and asymmetric internal pressure as environmental stress

Trunk growth, hormonally stimulated, provides an important *internal environment *in which scoliosis curves progress [96]. Likewise, asymmetric internal pressure of the intervertebral disc and vertebral growth plate in scoliosis suggests an *abnorma*l *stress environment *generates a positive feedback of cellular changes, resulting in curve progression due to a combination of factors [244,245]. These will include cyclical loads and asymmetrical changes in disc fluid content which affect vertebral growth (deforming three joint complex) [222].

#### Pathogenic asymmetry inducing and exacerbating processes

AIS asymmetry-inducing processes [238,240] - be they mechanical or biological - affecting vertebral growth plates, may render other factors including velocity of growth and hormones, abnormally increased or physiologic [3,6], to exacerbate the scoliotic deformity [243]. The etiologic, and potentially therapeutic, problem is to establish in each AIS girl, which process(es) in what pathway(s), is (are) abnormal, or exacerbating the deformity.

#### Longitudinal studies

In a cohort of normal individuals born in the UK in 1946 and surveyed longitudinally to the present, research is in hand to analyse tens of thousands of possible methylation sites in the DNA looking for changes that could explain the link between birth weight and breast cancer risk [246]. A similar study could evaluate AIS subjects.

### Epigenetics at the epicenter of modern medicine

Feinberg [17] writes:

*"Epigenetics, the study of non-DNA sequence-related heredity, is at the epicenter of modern medicine because it can help to explain the relationship between an individual's genetic background, the environment, aging, and disease..." *(see Appendix II).

The therapeutic potential of *epigenetics *for preventing and treating common human illness is threefold [17].

1) The possibility of new therapies because epigenetic changes are by definition reversible, unlike sequence mutations in disease.

2) Using medication to target biochemical pathways that are disturbed epigenetically in disease.

3) To intervene at the junction between genome and environment, to modify the effects of deleterious genes, and to influence the effects of the environment on phenotypic plasticity - ie, cells' ability to change their behavior in response to internal or external environmental cues.

The potential of these therapeutic and epigenetic epidemiologic approaches to AIS is at present unknown, and is restricted by the absence of established environmental factors involved in its etiopathogenesis.

### Network medicine and AIS

Barabasi [247] introduced the term *network medicine *which provides a network-based approach to human diseases by constructing *diseasomes*. According to Barabasi et al [248] given the functional interdependencies between the molecular components in a human cell, a disease is rarely a consequence of an abnormality in a single gene, but reflects the perturbations of the complex intracellular and intercellular network that links tissue and organ systems. *Interactome *describes the complex biological systems and cellular networks within cells [249].

Barabasi [247] states that network analysis is poised to play its biggest role at the cellular level since most cellular components are connected to each other through intricate regulatory metabolic and protein-protein interactions with proteomic assessment where research is needed [3,250]. The paradigm of network medicine is, *"think globally, act locally*".[248].

In AIS, consideration is needed for the possible creation of a network approach to etiopathogenesis by constructing AIS *diseasomes*.

### Summary and Conclusions

1. Genetic factors are believed to play an important role in the etiology of AIS in accordance with the *genetic variant hypothesis of disease*.

2. Sporadic reports, particularly for monozygotic twins but also other findings, suggest environmental factors are involved in the etiopathogenesis and phenotypic expression of AIS.

3. Research on the role of environmental factors and *epigenetics *has exploded in the last decade but not so for AIS (Figure 1).

4. Apart from the emerging role of epigenetic mechanisms in the etiology of neural tube defects [60], the Prader-Willi syndrome [71,72], and theoretical interpretations of Burwell and colleagues [73-76] and McMaster [77], *epigenetics *does not figure in any causal analysis of postnatal normal spinal growth, or in the etiopathogenesis of AIS (Figure 1).

5. There are three major ways organisms modify their DNAs inherited messages without changing DNA sequence: enzymes methylate DNA to modulate transcription; histone modifications and nucleosome positioning to induce or repress target sequences; and non-coding small RNAs to modify the expression of specific genes where there is therapeutic potential (Figure 3, Appendix V)

6. DNA methylation is an important epigenetic mechanism operating at the interface between genome and environment to regulate phenotypic plasticity with a complex regulation across the genome during the first decade of life

7. DNA methylation depends on dietary methionine and folate, both of which are affected by the nutritional state of the individual.

8. *Epigenetic modification *provides multicellular organisms with a system of normal gene regulation that silences portions of the genome and keeps them silent as tissues differentiate as *epigenotypes *(Figure 3).

9. Errors in this complex system from environmental and stochastic (random) events termed *epimutations *can give rise to abnormal gene silencing, that may result in a great deal of phenotypic variation and common disease.

10. *Epigenetic interactions *operating at cell, tissue, structure and organ levels, have been defined very recently by some workers in keeping with Waddington's inclusive definition of *epigeneticss; *the term is used to describe additional mechanisms for modulating cellular effects in response to changes in the internal and external environments without altering DNA sequence (Figure 4).

11. A molecular perspective encompassing *epigenetic modification and interactions *at vertebral growth plates for normal postnatal spinal growth and the etiopathogenesis of AIS is given.

12. The word *exposome*, means the totality of environmental exposures, external and internal, from conception onwards that create dysfunction leading in some individuals to occupational health problems,

13. The woed *exposome *is used here also in relation to physiologic and etiopathogenetic factors that respectively affect normal spinal growth and may induce the deformity of AIS namely, *physiologic growth-plate exposome and pathophysiologic scoliogenic exposome *(Figures 5 &6).

14. The concept of a *one-hit to multi-hit model *for AIS pathogenesis is mentioned.

### Future potential

1. The potential of epigenetic-based medical therapy for AIS cannot be assessed at present. It must await new research derived from the evaluation of epigenetic concepts for spinal growth in health and deformity.

2. Consideration is needed for the creation of a *network approach to *AIS etiopathogenesis by constructing AIS *diseasomes*,

3. These approaches, epigenetic and network, may possibly lead through several approaches - screening, genetic [195,198,199,251], epigenetic, biochemical [3,173-175,229,230], metabolic phenotypes [251] and pharmacogenomic [5,251], to the modulation of abnormal molecular pathways [108,179,252] by the development of novel preventive and curative measures based on diet, novel epigenetic drugs [13,108] and other approaches [52].

4. The tenets outlined here for AIS etiopathogenesis are applicable to other musculoskeletal growth disorders, including infantile and juvenile idiopathic scoliosis.

## Competing interests

The authors declare that they have no competing interests.

## Authors' contributions

GB with TG, PD and AM initiated this review at the time of presentations in London [240] and Padua [76]. GB wrote the text and conceived the novel concepts relating to the exposome and spinal development in health and AIS. PD and TG helped GB in finding and obtaining references. All authors contributed their professional skills to the ensuing discussions as the text and Figures progressed. All authors have read and approved the final manuscript.

## APPENDIX I

*Dual inheritance*. Holliday [58] points out that genetic inheritance in higher organisms normally refers to the transmission of information from one generation to the next. Nevertheless, there is also inheritance in somatic cells, characterized by the phenotypic stability of differentiated cells that divide (such as fibroblasts and lymphocytes), and also mitosis of stem line cells, which gives rise to another stem line daughter cell, and one that will differentiate.

## APPENDIX II

Feinberg [18] places *epigenetics *in perspective as follows (Figure 1):

"Traditionally, the pathology of human disease has been focused on microscopic examination of affected tissues, chemical and biochemical analysis of biopsy samples, other available samples of convenience, such as blood, and noninvasive or invasive imaging of varying complexity, in order to classify disease and illuminate its mechanistic basis. The molecular age has complemented this armamentarium with gene expression arrays and selective analysis of individual genes. However, we are entering a new era of epigenomic profiling, i.e., genome-scale analysis of cell-heritable nonsequence genetic change, such as DNAm. The epigenome offers access to stable measurements of cellular state and to biobanked material for large-scale epidemiological studies. Some of these genome-scale technologies are beginning to be applied to create the new field of epigenetic epidemiology."

According to Feinberg [18] the new filed of *epigenetic epidemiology *will measure and catalog epigenetic variation within and across populations in genome-scale analyses to characterize the correlation properties of methylation, similar to the catalog of SNP/CNV and linkage disequilibrium (non-random association of alleles at different loci), already showing promise in neuropsychiatric disease.

## APPENDIX III

*Phenotypic plasticity, time dependency and the CDGE, model for chronic disease*. A common theme to *disease epigenetics *is the disruption of *phenotypic plasticity*; this is the ability of cells to change their behavior in response to internal or external environmental cues over time [12,14-16]. Feinberg and colleagues [14,17,18] suggested the hypothesis that *epigenetics *provides an added layer of variation that might mediate the relationship between genotype and internal and external environmental factors which they termed the *common disease genetic and epigenetic hypothesis (CDGE*). This conjectural model overlies the *genetic hypothesis of disease *with an epigenetic component interacting with it [17,78]. *The CDGE, model *better explains the *age degeneration of epigenetic patterns *than does the *genetic hypothesis *[14].

*Genetic variant hypothesis of disease and non-genomic factors*. Butcher and Beck [1] write:

*"A spate of high-powered genome-wide association studies (GWAS) have recently identified numerous single-nucleotide polymorphisms (SNPs) robustly linked with complex disease. Despite interrogating the majority of common human variation, these SNPs only account for a small proportion of the phenotypic variance, which suggests genetic factors are acting in concert with non-genetic factors. Although environmental measures are logical covariants for genotype-phenotype investigations, another non-genetic intermediary exists: epigenetics." *[see [125]].

## APPENDIX IV

Haig [133] states that *epigenetics *has different meanings for different sciientists. Molecular biologists are familiar with the definition as:

*"The study of mitotically and/or meiotically heritable changes in gene function that cannot be explained by changes in DNA sequence *[135].

In contrast, functional morphologists would be more familiar with the definition:

*"... the entire series of interactions among cells and cell products which leads to morphogenesis and differentiation." *[136]. Herring continues, *"Thus all cranial development is epigenetic... Among the numerous epigenetic factors influencing the vertebrate face is mechanical loading. Loading seems to be particularly significant for formation and growth of skeletal tissues... Epigenetic influences range from hormones and growth factors to ambient temperature and orientation in a gravitational field." *[see [137-140]].

Feinberg [17] states that *epigeneticss *is at the heart of developmental biology, with the modern definition and Waddington's definition having converged. That is because "...the epigenetic state of an organism progresses from gamete to zygote to somatic tissue, all of which have profoundly different epigenomes, while the DNA is the same [18]. This view does not accommodate the concept of *epigenetic interactions *[137-140].

A few scientists take a more relaxed, or stricter view, either including RNA modification or limiting to vertical (generational) transmission [17].

*Epigenetics *does not invoke inheritance of mutational changes. leaving open what kinds of mechanism are at work [17]. An epigenetic system should be heritable, self-perpetuating and reversible [141]. Bird [25] wishing to avoid the constraints imposed by stringently requiring heritability in the definition of *epigenetics*, suggested the following:

"...the structural adaptation of chromosomal regions so as to register, signal or perpetuate altered activity states."

## APPENDIX V

*DNA methylation (*DNAm). The predominant epigenetic mechanisms involve DNA methylation, modifications to chromatin, genomic imprinting [106,155,156], and non-coding RNAs [46,52]. DNAm in mammals occurs almost exclusively as the covalent addition (or mark) of a methyl (CH_3_) group mainly to the nucleotide cytosine at cytosine-guanine dinucleotide sequences catalyzed by DNA methyltransferases (CpG islands, where 'p' indicates interstitial phosphate group between the DNA bases [10,17,26,29,63,141]). Promoters are key targets for epigenetic modification [63]. There are also covalent modifications of DNA-bound histones [157,158], notably acetylation, phosphorylation, methylation and ubiquitination [1,159-161]. Cytosine methylations of gene promoters which are reversible, are generally associated with silencing of genes, whereas histone acetylations are generally associated with activation of genes [26,99]. Many groups have studied the genomic distribution of DNA cytosine methylation and other chemical modifications of histone proteins to the *epigenome *[145,161]. Imprinting leads to the mono-allelic expression of certain genes depending on the parent origin of the allele controlled by *imprinting control regions *marked by DNA and histone methylation on one of the two parent alleles, perturbations of which induce diseases, including the Prader-Willi syndrome [52]. Recently it has become appreciated that hydroxymethylation of cytosine is a minor, but prevalent, form of base modification in addition to 5-methylation [162].

*DNAm and folic acid*. The source of methyl groups for DNAm is methionine an essential amino acid that is converted to a biologically active methyl donor state, S-adenosylmethionine, through a pathway involving folic acid, both of which are affected by the nutritional state [17,108,110, see Figure 1 of [108]]. Findings in subjects with chronic kidney disease and uremia have established a link between the epigenetic control of gene expression and xenobiotic influences, such as folate therapy [163]. Accoeding to Park et al [104] nutrients involved in one-carbon metabolism, namely folate, vitamin B12, vitamin B6, riboflavin, methionine, choline and betaine, are involved in DNA methylation by regulating levels of the universal methyl donor S-adenosylmethionine and methyltransferase inhibitor S-adenosylhomocysteine. Other nutrients and bioactive food components such as retinoic acid, resveratrol, curcumin, sulforaphane and tea polyphenols can modulate epigenetic patterns by altering the levels of S-adenosylmethionine and S-adenosylhomocysteine or directing the enzymes that catalyse DNA methylation and histone modifications.

*MicroRNAs, short interfering RNAs and potential for therapy*. MicroRNAs (miRNAs) are a class of short endogenous non-coding RNAs that act as post-transcriptional regulators of gene expression by attaching themselves to messenger RNA [52]. MiRNAs play fundamental roles in the control of many biological processes such as growth, development, differentiation and cell, death by repressing their target genes, and in relation to cancer and some other diseases [52]. Some miRNAs are regulated by epigenetic mechanisms, especially by methylation [52]. Short interfering RNAs, are a class of double-strnded RNA (dsRNA) molecules, that can guide methylation to complementary DNA, were first elucidated in plants, to enable the precise targeting of gene action [164]. *Plant*-*derived miRNAs *which enter the blood stream have been shown to muffle or amplify gene expression by binding to strands of messenger RNA with potential for therapy [53].

*DNAmn and metals. Epigenetics *may be the critical pathway by which metals produce their health effects [108,165]. Copper [166-168], zinc [168] and selenium [168,169] have each been linked to the pathogenesis of AIS. Other metals disrupt DNAm [170,171].

*DNAm in MZ twins, aging and epigenetic drift*. Recent studies using mostly peripheral blood lymphocytes (also skin, muscle and fat) and a battery of powerful molecular genetic methodologies coupled with competitive chromosomal hybridizations, suggest that phenotypic discordance between MZ twins is to some extent due to epigenetic factors that change over the lifetime of a multicellular organism [24,99]. It has been proposed that *epigenetic drift *during development can result from stochastic mechanisms (independent of environmental perturbations), or determined by such environmental perturbations [24,99,172]. Eckhardt et al [63] found no age-related DNAm change but did not report longitudinal data. In a longitudinal study, Bjornsson et al [14] found methylation changes over time with familial clustering suggesting that methylation maintenance may be under genetic control. In 46 MZ twin-pairs and 45 DZ twin-pairs Wong et al [50] found that DNAm differences are apparent already in early childhood, even between genetically identical individuals; and that individual differences in methylation are not stable over time. Unlike the primary DNA sequence, methylation status will depend on the tissue being analysed [Armour J personal communication]. Epigenetic mechanisms may be causal in the aging process and be influenced by diet providing opportunities to improve health in later life [108].

## APPENDIX VI

In connection with *epigenetic interactions in normal development*, Lieberman [138] writes:

"In normal development, " .... hormones and growth factors bind with specific receptors in the cell membrane or nucleus. Activated receptors then trigger a cellular response through mechanisms such as altering gene transcription, altering ion transport in and out of the cell, activating or inhibiting intracellular enzymes, stimulating protein synthesis, or inducing cellular proliferation."

"Regulation of local growth occurs through interactions between the genes that cause skeletogenic cells to synthesize, resorb, or otherwise modify skeletal tissue, and stimuli from other genes or cells. Such interaction between cells and their environment (which includes other cells) are generally categorized as epigenetic interactions... Additional categories of epigenetic interactions influencing morphogenesis include systemic hormones, growth factors, and the effects of mechanical loading."

*"Bones do have a strong genetic component to their growth and development, but a set of complex and constrained interactions between bone cells and their mechanical environment can influence bone morphology, particularly while the skeleton is still growing." *(see Herring [136] in Appendix IV]).

## APPENDIX VII

*Some hypotheses and concepts of AIS etiopathogenesis *[2,6,192]

(1) Genetics [2,4,5,92,193-199].

(2) Biomechanical spinal growth modulation [181,182].

(3) Relative anterior spinal overgrowth (RASO) [200-203].

(4) Dorsal shear forces and axial rotation instability [204,205].

(5) Asynchronous spinal neuro-osseous growth [206-211].

(6) Postural abnormalities including vestibular and CNS dysfunction [2,212,213].

(7) Motor control problem [214-217].

(8) Body-spatial orientation concept [218].

(9) Neurodevelopmental concept [219].

(10) Thoracospinal concept [79-83,220,221].

(11) Deforming three joint complex hypothesis [222].

(12) Systemic melatonin deficiency [223-226].

(13) Systemic melatonin-signaling pathway dysfunction [173,174,177,227-230].

(14) Relative osteopenia [113,114,231,232].

(15) Systemic platelet calmodulin dysfunction [233-236].

(16) Developmental instability & symmetry control dysfunction [85-87,237-241].

(17) Intrinsic growth plate asymmetry hypothesis [74,75,188,237-241].

(18) Collective and escalator models [192].

(19) Leptin-hypothalamic-sympathetic nervous system *(LHS) *dysfunction with disharmony between somatic and autonomic nervous systems in the spine and trunk [[6], see [3,242]].
